# Identification of a New Variety of Avocados (*Persea americana* Mill. CV. Bacon) with High Vitamin E and Impact of Cold Storage on Tocochromanols Composition

**DOI:** 10.3390/antiox9050403

**Published:** 2020-05-09

**Authors:** Celia Vincent, Tania Mesa, Sergi Munne-Bosch

**Affiliations:** 1 Department of Evolutionary Biology, Ecology and Environmental Sciences, University of Barcelona, Faculty of Biology, Av. Diagonal 643, E-08028 Barcelona, Spain; cevisa96@gmail.com (C.V.); tmesapar7@alumnes.ub.edu (T.M.); 2 Research Institute of Nutrition and Food Safety (INSA), University of Barcelona, Faculty of Biology, Av. Diagonal 643, E-08028 Barcelona, Spain

**Keywords:** avocados (*Persea americana* Mill.), low temperatures, plastochromanol-8, tocotrienols, tocopherols, tocochromanols

## Abstract

(1) Background: Tocochromanols are a group of fat-soluble compounds including vitamin E (tocopherols and tocotrienols) and plastochromanol-8, and just one avocado can contain up to 20% of the required vitamin E daily intake. (2) Methods: HPLC and LC-MS/MS analyses were performed in avocados of various varieties and origin for the identification and quantification of tocopherols, tocotrienols and plastochromanol-8. After selection of the variety with the highest vitamin E content, we evaluated to what extent short- (4 h) and long-term (10 d) cold storage influences the accumulation of tocochromanols. (3) Results: Analyses revealed that “Bacon” avocados (*Persea americana* Mill. cv. Bacon) were the richest in vitamin E compared to other avocado varieties (including the highly commercialized Hass variety), and they not only accumulated tocopherols (with 110 µg of α-tocopherol per g dry matter), but also tocotrienols (mostly in the form of γ-tocotrienol, with 3 µg per g dry matter) and plastochromanol-8 (4.5 µg per g dry matter). While short-term cold shock did not negatively influence α-tocopherol contents, it increased those of γ-tocopherol, γ-tocotrienol, and plastochromanol-8 and decreased those of δ-tocotrienol. Furthermore, storage of Bacon avocados for 10 d led to a 20% decrease in the contents of α-tocopherol, whereas the contents of other tocopherols, tocotrienols and plastochromanol-8 were not affected. (4) Conclusions: It is concluded that Bacon avocados (i) are very rich in α-tocopherol, (ii) not only contain tocopherols, but also tocotrienols and plastochromanol-8, and (iii) their nutritional vitamin E value is negatively influenced by long-term cold storage.

## 1. Introduction

Tocochromanols are a group of amphiphilic molecules that includes tocopherols, tocotrienols and plastochromanol-8 [1,2]. These are all composed by a polar chromanol head and a highly apolar polyprenyl side chain that provide them with the capacity to exert an antioxidant function in membranes, from cyanobacteria and plants where they are synthetized until a variety of tissues in animals and humans, which incorporate tocochromanols regularly from their daily dietary intake [1,2]. While tocopherols have a saturated phytyl-derived side chain, tocotrienols and plastochromanol-8 tails are more unsaturated since they derive from geranylgeranyl-diphospate and solanesyl-diphospate, respectively [3]. Both tocopherols and tocotrienols include various homologues according to the position and methylation degree in the chromanol head, thus identifying four different molecules for each group (α-, β-, γ- and δ-tocopherols and -tocotrienols). All tocochromanols exert an efficient antioxidant activity by inhibiting the propagation of lipid peroxidation through scavenging lipid peroxyl radicals and by preventing it through the (physical) quenching and (chemical) scavenging of singlet oxygen, not only in plant tissues where they are synthetized, but also in humans, although such a role is mainly generally attributed to α-tocopherol in the human body, since this is the major form transported by a specific protein [4]. 

While α-tocopherol is a universal molecule found in plants, animals and humans, tocotrienols have not been described in all photosynthetic tissues; rather, they accumulate in seeds and fruits of some plant species only [5,6]. Similarly, plastochromanol-8 is not present universally, despite being found in many plant tissues such as seeds, leaves, buds, flowers and fruits of several species [2,7,8]. Interestingly, in vitro studies in hydrophobic solvents show a higher antioxidant activity against singlet oxygen for tocotrienols and plastochromanol-8 than for tocopherols, which has been attributed to their more apolar structure of the side chain [2]. New nutraceutical functions have been recently attributed to tocotrienols. For instance, antiangiogenic properties against osteoporosis, atherosclerosis, inflammatory processes and many types of cancer (like colorectal, prostate, lung and pancreas cancer) have been reported, mainly for δ- and γ-tocotrienol forms [9]. Otherwise, despite not being considered a molecule belonging to the vitamin E family, the antioxidant activity of plastochromanol-8 is of great relevance in plants and it may also probably display beneficial properties in humans, although to our knowledge this has not been studied thus far in detail.

Cold storage of fruits is an effective means to prevent food deterioration, particularly in climacteric fruits such as avocados (*Persea americana* Mill), where low temperatures reduce ethylene production and therefore inhibit fruit over-ripening [10,11]. Indeed, introducing avocado fruits in cold chambers is a common technique implemented for preventing their early deterioration, thus increasing fruit marketability. However, the cellular redox balance in fruits may be threatened by the extent of low temperature exposure in storage chambers before fruits reach the consumers. Indeed, low temperature shocks or long-term cold exposure can cause a loss of cellular antioxidant defenses in fruits [12,13,14]. As a result, oxidative reactions may occur in an uncontrolled manner resulting in sustained oxidative stress and tissue damage [13]. To fight against oxidation reactions at low temperatures, tocopherols, mainly α-tocopherol, have been suggested to be essential for attaining acclimation in plants [3]. Additionally, some studies have reported that tocotrienols may be as effective as tocopherols in protecting leaves from photooxidation processes under low temperatures [1]. Although several studies have been performed in leaves of *Arabidopsis* and other model plant species to link tocopherol accumulation and low temperature acclimation [15,16,17,18], very few studies have investigated thus far the influence of cold temperature storage on the accumulation of tocochromanols in fruits; except for an increase in tocopherols upon 3 d of storage at cold temperatures in sweet cherries, which is a fruit with low concentrations of fatty acids and vitamin E [19] and a maintenance of constant α-tocopherol contents in oil of Fuerte avocados upon exposure to 5 °C for three weeks [20]. To our knowledge, the effects of low temperatures on the accumulation of tocochromanols in other avocado varieties, and of tocotrienols and plastochromanol-8 in fruits in general have not been investigated thus far. 

Avocados (*Persea americana* Mill) are highly valuable fruits with increasing interest for consumers thanks to their nutraceutical properties due to antioxidant contents, such as ascorbate (vitamin C, 8.8 mg/100 g) and B-type vitamins (such as vitamin B_6_, 0.29 mg/100 g), fiber (6.8 g/100 g), phytosterols (83.1 mg/100 g), monounsaturated fatty acids (9.8 g/100 g) and vitamin E (2.36 mg/100 g) [21]. These are originally from Mexico where tropical environmental conditions permitted hybridization techniques that have led to the wide number of varieties currently found worldwide [22]. Nevertheless, the main producing countries are still those with warm climates which export a huge percentage of their production to other continents [23]. Hass is the most widely produced and commercialized avocado variety worldwide, while Bacon, a hybrid variety originally cultivated in 1954 by James Bacon in California, occupies the third position (after Hass and Fuerte) in terms of agricultural production in Spain, the main avocado producer in Europe [24]. Here, avocado fruits (*Persea americana* Mill) were investigated aiming to determine (i) the amounts and composition of tocochromanols in the edible part of various avocado varieties and (ii) how cold storage in the short and long term influences tocochromanol contents in Bacon avocados. This study shows to what extent cold storage implemented along the supply chain can negatively influence the nutritional quality of avocados in terms of vitamin E accumulation.

## 2. Material and Methods

### 2.1. Plant Material and Samplings

Avocados (*Persea americana* Mill.), either collected at commercial harvest maturity in the field or purchased from supermarkets in a non-ripe stage (depending on the experiments, see details below), were immediately brought to the laboratory at the University of Barcelona (Barcelona, NE Spain) and used for assays. In all cases, fruits were selected for homogeneity according to their size and lack of pathogen symptoms. Three independent experiments were performed.

For the identification of tocochromanols in various avocado varieties and origins (experiment 1), fruits were purchased in local supermarkets or markets, as follows. Non-ripe Hass avocados originated from Brazil, Perú and Spain, and identified as such in their label, were obtained from a supermarket in Barcelona (NE Spain) and immediately transported at room temperature by car to the laboratory. Non-ripe Hass and Fuerte avocados from Chile and Govín avocados from Cuba were obtained from local markets and transported by plane and car to the laboratory. Finally, Bacon avocados were obtained from a commercial orchard in Málaga (south Spain) at a mature stage and brought to the laboratory after 12 h of transportation at 8–10 °C. All fruits were then exposed to room temperature in the laboratory at the University of Barcelona and when firmness attained levels of 3N for all fruits from all varieties and origins, then the mesocarp tissue of four fruits per variety and origin was sampled and immediately frozen in liquid nitrogen and stored at −80 °C until analyses. With these samples, tocochromanols were quantified by high-performance liquid chromatography (HPLC) and identification of compounds confirmed by liquid chromatography coupled to electrospray ionization mass spectrometry in tandem (LC-ESI-MS/MS). 

The influence of short-term, cold shock exposure on tocochromanol accumulation in Bacon avocados (experiment 2) was examined by performing samplings just before and after 4 h of cold shock of fruits at 4 °C in a cold storage chamber (Frimatic, S.A., Barcelona, Spain). Samples from 18 randomly selected fruits at each time point including 0 h and 4 h were immediately frozen in liquid nitrogen and stored at −80 °C until analyses. With these samples, tocochromanols were quantified by HPLC while the extent of lipid peroxidation and changes in photosynthetic pigments were estimated spectrophotometrically, as described below.

The influence of long-term exposure to low temperatures on tocochromanol accumulation in Bacon avocados (experiment 3) was examined by performing samplings just before and during exposure for a period of 10 d of cold storage of fruits at 4 °C using the same cold chamber (Frimatic, S.A.). Mesocarp samples from 18 randomly selected fruits at times including 0 d, 2 d, 5 d, 7 d and 10 d of low temperature exposure were immediately frozen in liquid nitrogen and stored at −80 °C until analyses. With these samples, tocochromanols were quantified by HPLC while the extent of lipid peroxidation and changes in photosynthetic pigments were estimated spectrophotometrically, as described below.

### 2.2. Tocochromanol Analyses

The quantification of the different tocochromanol forms, including tocopherols, tocotrienols and plastochromanol-8, was performed as described previously [25] with some modifications. One-hundred mg of avocado (mesocarp) sample was extracted with 1 mL of methanol containing 0.01% (*w/v*) butyl-hydroxytoluene (BHT) and 5 ppm (*w/v*) of tocol as an internal standard. Extraction was performed using 30 min of ultrasonication (Bransonic ultrasonic bath 2800, Emerson Industrial, Danbury, CT, USA) just after vortexing for 20 s. Then, samples were centrifuged at 600 *g* during 10 min at 4 °C to subsequently recover supernatants with a hydrophobic PTFE filter 0.22 μm (Phenomenex, Torrance, CA, USA). Tocochromanols were separated by HPLC at room temperature using an Inertsil 100A column (5 μm, 30 × 250 mm, GL Sciences Inc., Tokyo, Japan). Quantification was performed using a Jasco fluorescence detector (FP-1520, Tokyo, Japan) and a calibration curve established with each of the tocochromanols analyzed and corrected with the tocol recovery, which was always above 97%. 

The identification of tocochromanols was confirmed by using high-performance liquid chromatography coupled to electrospray ionization mass spectrometry in tandem (LC-ESI-MS/MS) as described previously [26]. Methanolic extracts were obtained as described before for the HPLC analyses and used here for the identification of tocochromanols by LC-ESI-MS/MS. Tocochromanol separation was performed with an Inertsil 100A column (5 μm, 30 × 250 mm, GL Sciences Inc. (Tokyo, Japan), and an isocratic flow of hexane:dioxane (95.5:4.5 *v/v*) mobile phase. The MS acquisition was performed using negative ionization between *m/z* 100 and 650, with the Turbo Ionspray source. In addition, quadrupole time-of-flight (QqToF) mass spectrometry was used to obtain product ion information. The MS parameters were: ion spray voltage, −4200; declustering potential (DP), −40; focusing potential (FP), −150; declustering potential two (DP2), −10; ion release delay (IRD), 6 V; ion release width (IRW), 5 ms; nebulizer gas, 50 (arbitrary units); curtain gas, 60 (arbitrary units), and auxiliary gas N_2_, 6000 cm^3^ min^−1^ heated at 500 °C. 

### 2.3. Lipid Peroxidation Assays

To determine the extent of lipid peroxidation, primary (lipid hydroperoxide) and secondary (malondialdehyde, MDA) lipid peroxidation products were analyzed, as follows. For lipid hydroperoxides analyses, frozen samples (100 mg) were repeatedly (three times) extracted with 1 mL methanol + 0.01% BHT (*w/v*) at 4 °C using 30 min of ultrasonication (Bransonic ultrasonic bath 2800). After centrifugation, supernatants were collected, combined and used for analyses using the Fox-2 reagent (consisting in a solution of 90% methanol (*v/v*) containing 25 mM sulfuric acid, 4 mM butylhydroxyltoulene (BHT), 250 μM iron sulfate ammonium (II) and 10 μM xylenol orange) as described in Bou et al. [27]. Absorbances were measured at 560 nm and 800 nm. A calibration curve using hydrogen peroxide 37% (*v/v*) was used for quantification.

For estimation of the MDA content, the thiobarbituric acid-reactive substances (TBARS) assay, which considers the possible influence of interfering compounds, was used [28]. In short, 100 mg of sample was extracted with 3 mL of ethanol 80% (*v/v*) containing 0.01% (*w/v*) BHT, vortexed for 20 s and exposed to ultrasonication for 15 min (Bransonic ultrasonic bath 2800). After centrifuging at room temperature for 13 min at 600 *g*, the supernatant was recovered, and the pellet re-extracted twice using the same procedure. Then, two tubes were used: (a) − TBA, with 1 mL extract + 1 mL 20% trichloroacetic acid (*w/v*) with 0.01% BHT (*w/v*) and (b) + TBA, with 1 mL extract + 1 mL 20% trichloroacetic acid (*w/v*), 0.01% BHT (*w/v*) and 0.65% thiobarbutiric acid (*w/v*). Tubes were incubated for 25 min at 95 °C and then the reaction was stopped by maintaining them at 4 °C for 10 min. After centrifugation at 600 *g* at room temperature for 5 min, MDA content in samples were analyzed by spectrophotometry at 440, 532 and 600 nm and quantified using the equations developed by Hodges et al. [28].

### 2.4. Chlorophyll Content

To determine total chlorophyll content, samples (100 mg) were extracted in 1 mL of methanol + 0.01% BHT as explained before, using vortex and ultrasonication for 30 min at 4 °C. Supernatants were collected after centrifugation for 10 min at 600 *g* and 4 °C. Chlorophylls were measured spectrophotometrically reading absorbances at 653, 666 and 750 nm and measuring chlorophyll content as described [29].

### 2.5. Statistical Analyses

Statistical analyses were performed by one-way ANOVA and Tukey posthoc tests were used for multiple comparisons among time (IBS SPSS Statistics 19; SPSS Inc., Chicago, IL., USA). Differences were considered significant when *p* values were under the significance level α = 0.05. 

## 3. Results

### 3.1. Identification of Tocochromanols in Various Avocado Varieties

In order to determine the presence and amount of tocochromanols in the mesocarp (edible part of the fruit) of different avocado varieties, HPLC and LC-ESI MS/MS analyses were performed. Among the various varieties and origins tested, Bacon avocados from Spain showed the largest amount of vitamin E (Table 1A). Bacon avocados contained 2.4 mg α-tocopherol per 100 g of edible fruit, which coincided with a very low quantity of its precursor γ-tocopherol in the tissue (Table 1A). Bacon was the variety with the highest tocochromanol content among all studied varieties. By contrast, Govín from Cuba, Hass from Brazil and Hass from Perú were the avocados showing the lowest amounts of total tocochromanols. 

A comparison of four origins of Hass avocados (Chile, Spain, Perú and Brazil) revealed that the origin had a very strong effect on tocochromanol contents, including α-tocopherol (Table 1). All avocado varieties behaved similarly to Bacon avocados from Spain in terms of accumulating most of the tocochromanols in the form of α-tocopherol but both Bacon from Spain and Govín from Cuba presented a larger amount of α-tocopherol than the highly commercialized Hass variety irrespective of its origin. Furthermore, although plastochromanol-8 was present in all avocado varieties, its contents were higher in Hass varieties (irrespective of the origin) than in Bacon and Govín (from Spain and Cuba, respectively). Notably, δ-tocotrienol seemed to be exclusively present in Bacon (Table 1A). Results differed slightly when comparing the vitamin E amounts per unit of dry weight in different varieties; Bacon occupied the second position in terms of vitamin E accumulation, just after Govín, as the contents of α-tocopherol were higher in these two varieties than in Hass or Fuerte (Table 1B). Moreover, total tocochromanol contents were also higher in Bacon when compared to the other highly commercialized varieties, except for Govín which occupied the first position just before Bacon variety (Table 1B).

**Figure 1:**
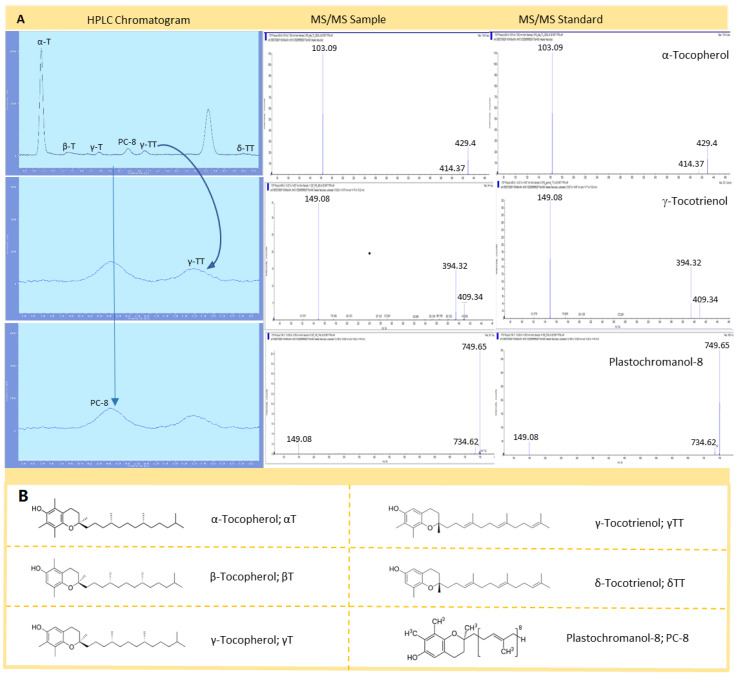
(**A**): Separation (by HPLC, *left*) and identification (by liquid chromatography coupled to electrospray ionization mass spectrometry in tandem [LC-ESI-MS/MS], *center* and *right*) of tocochromanols in Bacon avocados. (**B**): Chemical formula of tocochromanols identified in Bacon avocados.

The major tocochromanol present in the mesocarp (edible tissue) of Bacon avocados was α-tocopherol (with an 87.8%), as clearly observed in the HPLC chromatogram (Figure 1A), followed by plastrochromanol-8, β- and γ-tocopherols, and δ- and γ-tocotrienols (Table 1A). HPLC identification by retention time was confirmed by LC-ESI MS/MS using the corresponding authentic standards, which showed exactly the same fragmentation patterns as the corresponding peaks in the samples (Figure 1). This tocochromanol profile in Bacon avocados, enriched in the α-tocopherol form and with the presence of δ-tocotrienol, is different from that found for the Hass variety (Table 1, see also [30,31]).

### 3.2. Cold-induced Changes in Tocochromanol Composition in Bacon Avocados

**Figure 2:**
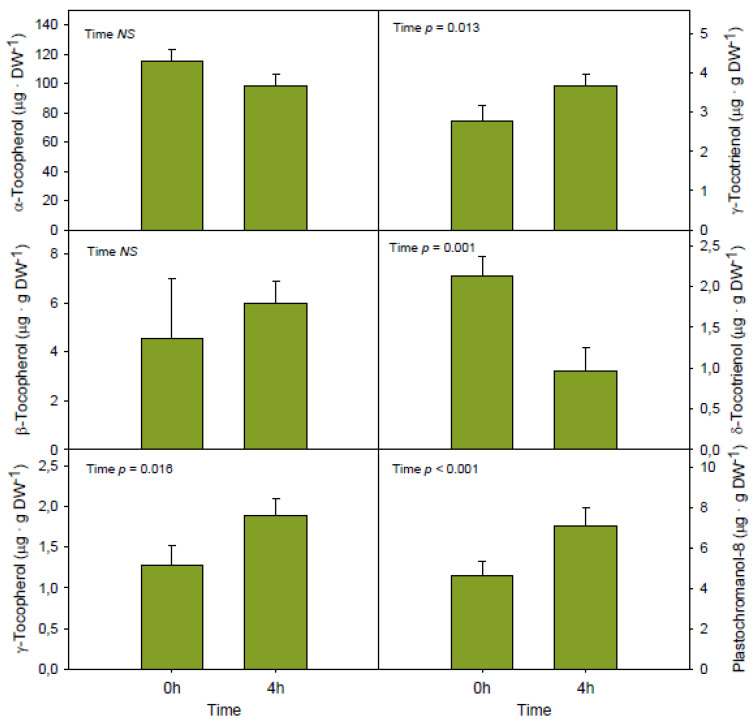
Influence of short-term (4 h) exposure of Bacon avocados to cold temperatures (4 °C) in the contents of tocochromanols. Data represent the mean ± SE of *n* = 18 fruits. Differences were considered significant when *p* < 0.05. DW, dry weight.

**Figure 3:**
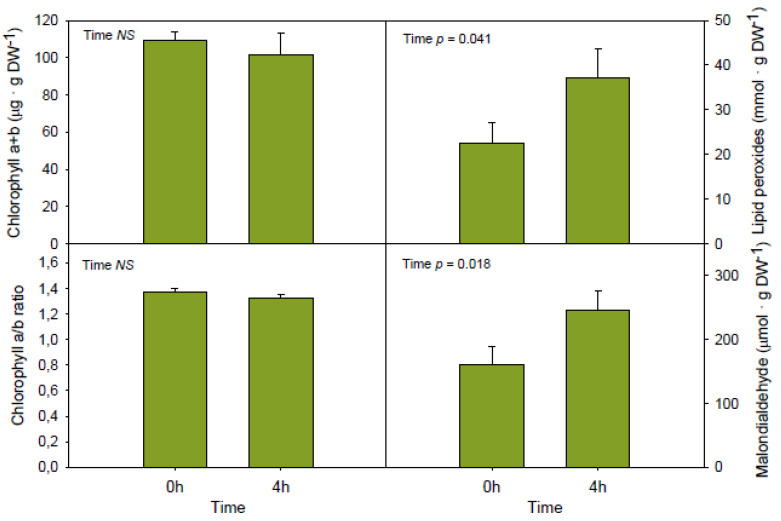
Influence of short-term (4 h) exposure of Bacon avocados to cold temperatures (4 °C) in the contents of chlorophylls, chlorophyll a/b ratio and the extent of lipid peroxidation (estimated as the contents of lipid hydroperoxides and malondialdehyde, as indicators of primary and secondary lipid peroxidation, respectively). Data represent the mean ± SE of *n* = 18 fruits. Differences were considered significant when *p* < 0.05. DW, dry weight.

After a low temperature shock for 4h, the contents of the major tocochromanol present in the mesocarp of Bacon avocados, α-tocopherol, were not altered (Figure 2). The same was observed for β-tocopherol, but not for the other tocochromanols. While the contents of plastochromanol-8, γ-tocopherol and γ-tocotrienol increased, those of δ-tocotrienol decreased, with the latter showing a reduction by 60% under cold treatment (Figure 2). This cold-induced shift in the tocochromanol composition was accompanied by an increase in the extent of lipid peroxidation, as indicated by 60% increases in lipid hydroperoxides and malondialdehyde contents, while chlorophyll levels and the chlorophyll a/b ratio remained unaltered (Figure 3). 

**Figure 4:**
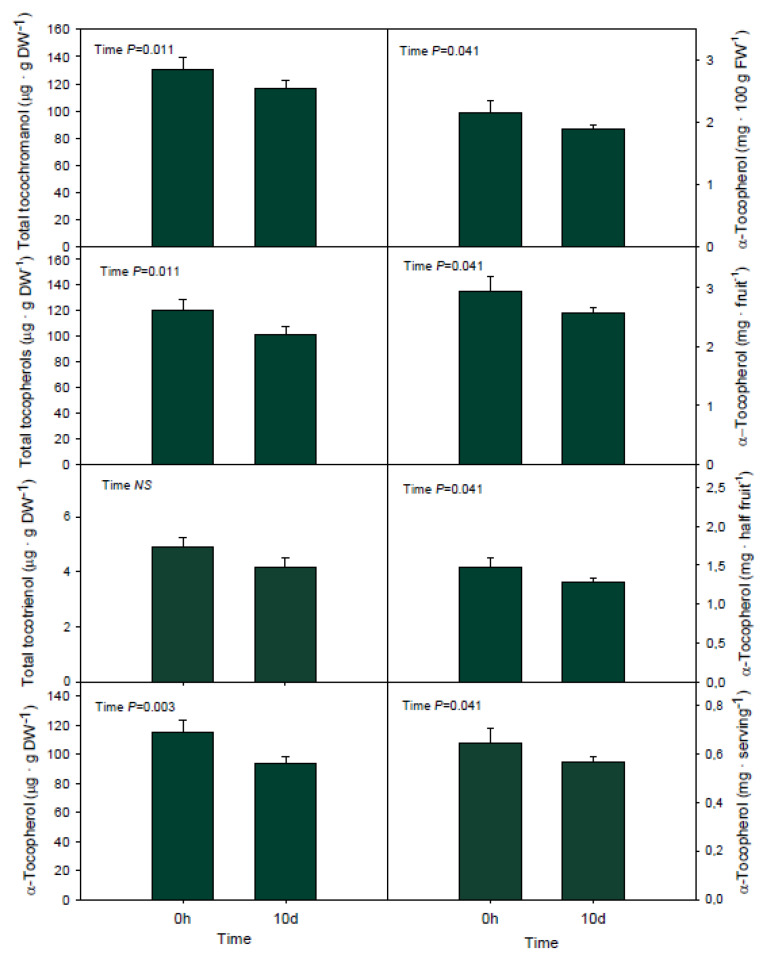
Influence of long-term (10 d) storage of Bacon avocados at cold temperatures (4 °C) in the contents of total tocochromanols, tocopherols, tocotrienols and α-tocoperol. Contents of α-tocopherol are also shown in mg per 100 g of mesocarp in fresh weight (FW), per one fruit (136 g FW), per a half (68 g FW) and per serving according to the Nutrition Labelling and Education Act (NLEA; corresponding to 30 g FW). Data represent the mean ± standard error of *n* = 18 fruits. Differences were considered significant when *p* < 0.05. DW, dry weight.

Total tocochromanol contents showed a decrease by 16% after 10 d of storage at low temperatures, which was mostly due to a significant decrease in tocopherols but not tocotrienols (Figure 4). Part of this loss was related to the decrease in the major tocochromanol form in Bacon avocados, α-tocopherol, which decreased by 20% after 10 d of cold storage (Figure 4). When α-tocopherol contents were expressed on a fresh weight basis (either per 100 g FW, per fruit, half fruit or serving), a decrease in its contents was also observed, thus offering a lower amount (by 15%) of α-tocopherol per amount of fruit consumed (Figure 4). This reduction in vitamin E contents occurred progressively over time, as revealed by the time-course evolution of α-tocopherol contents (Figure S1), but most particularly between 5 d and 10 d of cold storage. In contrast to short-term exposure to cold temperature, the other tocochromanol forms were not clearly affected by long-term cold storage, although γ-tocopherol and δ-tocotrienol showed slight variations over time (Figure S1). Reductions of α-tocopherol during long-term storage at low temperatures was coincident with a 3.4-fold increase in malondialdehyde contents after 10 d of cold storage (Figure S2).

## 4. Discussion

### 4.1. Bacon is a Variety of Avocados with High Tocochromanol Contents

Among the avocado varieties examined in our study, Bacon from Spain was the one showing the highest vitamin E content. All varieties showed a similar tocochromanol composition, so that the major tocochromanol was α-tocopherol, except Bacon, which also accumulated some amounts of δ-tocotrienol. Hence, tocochromanols composition was in general enriched in α-tocopherol, with a diminished accumulation of other tocopherols and tocotrienols, with the overall most notable exception of γ-tocochromanols in all varieties and additionally of δ-tocotrienol in Bacon. Varietal differences might be associated not only with the geographical origin of the fruit, as shown in our results, but also with the highly heterozygous genetic origin of avocado races. *Persea americana* includes *P. americana var. drymifolia* (commonly known as Mexican race)*, var. guatemalensis* (known as Guatemalan race) and *var. americana* (or West Indian race). While Bacon is obtained from the hybridization of Mexican x Guatemalan races, Hass variety is generally reported to have a pure Guatemalan origin [32]. However, breeding strategies, cross- and self-pollination techniques, different strategies of cultivation and the posterior selection according to farmer preferences, like high yield, fruit quality and long shelf life, usually give rise to quite heterogenic crops in the same variety, which might lead to the observed differences in the Hass avocados from different origins studied here that showed notable differences in the accumulation of tocochromanols.

Plastochromanol-8 accumulation in fruits may be of particular relevance since this is also a powerful antioxidant, even showing higher antioxidant activity than α-tocopherol in hydrophobic environments due to its more highly unsaturated prenyl chain [2]. Plastochromanol-8 was found in mesocarp tissue of avocado fruit at relatively low amounts compared to α-tocopherol, but some differences between cultivars were observed. In this case, Hass was the variety with the greatest amount of this compound compared to the other studied varieties, including Bacon. Furthermore, we reported here on the accumulation of tocotrienols in avocados, which contrasts with a recent report [8] showing the accumulation of plastochromanol-8 but not of tocotrienols in Hass avocado. Our study shows that tocotrienols may accumulate in avocado fruits, in particular in some varieties such as Bacon. Notably, δ-tocotrienol was only found in Bacon among all studied varieties. Beneficial properties for humans have recently been attributed to this compound, in particular to help in the prevention of the development of various cancers, including breast, colorectal, lung and many other types of cancer, apart from providing anticholesterolemic and antidiabetic benefits [33,34,35]. Although the contents of δ-and γ-tocotrienols were relatively low compared to that of α-tocopherol in Bacon avocados, these compounds might, to some extent, exert an additional beneficial response in the human body, an aspect that deserves further investigations.

### 4.2. Effects of Short and Long-Term Storage on Tocochromanol Contents

While a low temperature shock for 4 h did not alter α-tocopherol contents in the mesocarp of Bacon avocados, long-term storage for 10 d led to significant decreases in vitamin E contents. In contrast to unaltered contents of tocopherols after 4 h of low temperature exposure, the contents of plastochromanol-8, γ-tocopherol and γ-tocotrienol increased, and those of δ-tocotrienol decreased, the latter showing a reduction by 60% under cold treatment. Therefore, cold shock led to significant reductions in the levels of δ-tocotrienol and the cold-induced shift in the tocochromanol composition was accompanied by an increase in the extent of lipid peroxidation, as indicated by 60% increases in lipid hydroperoxides and malondialdehyde contents. Interestingly, chlorophyll contents were unaltered during the same period and Bacon avocados stored for 5d did not show alterations in the extent of lipid peroxidation, as indicated by the same measurements. This suggests that the cold-induced shift in tocochromanol composition was mainly due to metabolic alterations that resulted in transient lipid peroxidation, but this was not accompanied with a quality loss. In contrast, α-tocopherol contents decreased during long-term storage of Bacon avocado fruits at low temperatures, a decrease that was accompanied by an increase in the extent of lipid peroxidation, which was reflected by an increase in malondialdehyde. This result contrasts with a previous study [20] showing that α-tocopherol in oil obtained from Fuerte avocados keeps stable after 3 weeks of storage at 5 °C. This difference may be due to different reasons, including not only the study of a different variety (Fuerte in [20] and Bacon in our study), but also to a higher stability of α-tocopherol in oil at 5 °C [20] than in entire fruits at 4 °C in our study. Unfortunately, little research has been performed thus far to evaluate how cold storage temperature influences tocochromanol composition in vitamin E-rich fruits and further studies are required to better understand the causes of vitamin E instability in avocado fruits and oils.

### 4.3. Balance between Storage and Nutritional Value

According to the Nutritional Labelling and Education Act (NLEA) and the National Health and Nutrition Examination Survey (NHANES), the serving of avocado is recommended to be of 30 g or half of an avocado, respectively, which corresponds to an intake of 0.59 mg and 1.34 mg of α-tocopherol, respectively. Interestingly, when the nutritional value in terms of vitamin E was measured over time of cold storage, a decrease in tocochromanols occurred, which was mostly attributed to a progressive drop in α-tocopherol content, so that the daily vitamin E intake is significantly lower if avocados are stored for 10 d at 4 °C. In contrast, other tocochromanols were not affected. According to the results presented in our study, avocados stored for 10 d at low temperatures start to suffer oxidation processes, which might be related to the stress situation experienced by the mesocarp due to cold temperatures, which can lead to oxidative damage. Indeed, α-tocopherol levels dropped up to 20% after 10 d of cold storage, and this loss of α-tocopherol contents may slightly contribute to a lesser intake and absorption of vitamin E in the human diet under the levels of reference [36]. Furthermore, the loss in detoxifying oxygen radicals function by a loss of antioxidants such as vitamin E due to cold storage of fruits for long periods may contribute to a higher risk of suffering from cardiovascular diseases like atherosclerosis, cancer and cataracts, among other diseases related to degenerative processes [37,38,39,40]. 

## 5. Conclusions

In conclusion, Bacon has been shown to be the variety with very high tocochromanol contents relative to other studied varieties, presenting values greater than those of the highly commercialized Hass variety. Furthermore, Bacon variety marketing should be fostered not only because of the high amounts of vitamin E but also because it was the only variety showing δ-tocotrienol, a compound that might have additional beneficial effects. Moreover, according to procedures implemented along the supply chain which consist of introducing fruits into cold chambers, our study showed that 10 d might be the threshold where cold stress is starting to induce losses in vitamin E, hence decreasing nutritional value and fruit quality.

## Figures and Tables

**Table 1:** Contents of tocochromanols in different avocado varieties, including various origins.

**(A) Tocochromanol (µg/100 g FW)**							
	**α-T**	**β-T**	**γ-T**	**γ-TT**	**δ-TT**	**PC-8**	**Total TCs**
Bacon Spain	2371 ± 148^c^	140.3 ± 22.7^d^	50.3 ± 11.2	42.2 ± 3.3	47.7 ± 5.7	191 ± 23^b^	2848 ± 181^c^
Fuerte Chile	2190 ± 52^bc^	79.8 ± 4.9^bcd^	29.9 ± 4.5	32.1 ± 5.0	ND	192 ± 17^b^	2524 ± 53^abc^
Hass Chile	2068 ± 48^bc^	88.4 ± 7.6^cd^	51.4 ± 5.1	27.4 ± 3.8	ND	300 ± 18^cd^	2535 ± 61^bc^
Govín Cuba	2004 ± 129^bc^	ND	25.5 ± 5.5	29.6 ± 1.8	ND	93 ± 4^a^	2152 ± 133^a^
Hass Spain	1997 ± 211^bc^	57.9 ± 8.2^abc^	50.5 ± 4.3	39.2 ± 3.7	ND	284 ± 21^c^	2434 ± 296^abc^
Hass Perú	1592 ± 225^b^	14.1 ± 8.2^a^	36.4 ± 3.4	32.1 ± 11.5	ND	378 ± 22^d^	2062 ± 224^ab^
Hass Brazil	816 ± 209^a^	18.0 ± 6.8^ab^	31.8 ± 4.7	42.9 ± 6.3	ND	226 ± 12^b^	1138 ± 217^ab^
**(B) Tocochromanol (µg/g DW)**							
	**α-T**	**β-T**	**γ-T**	**γ-TT**	**δ-TT**	**PC-8**	**Total TCs**
Bacon Spain	127.3 ± 10.7^c^	7.5 ± 1.2^b^	2.7 ± 0.6^b^	2.2 ± 0.1^bc^	2.5 ± 0.3	10.3 ± 1.4^b^	153 ± 13^b^
Fuerte Chile	47.3 ± 4.8^ab^	1.7 ± 0.1^a^	0.6 ± 0.1^a^	0.7 ± 0.1^a^	ND	4.2 ± 0.7^a^	54 ± 5^a^
Hass Chile	60.4 ± 1.6^ab^	2.6 ± 0.3^a^	1.5 ± 0.2^ab^	0.8 ± 0.1^a^	ND	8.8 ± 0.6^ab^	74 ± 2^a^
Govín Cuba	174.2 ± 16.2^d^	ND	2.2 ± 0.4^a^	2.6 ± 0.2^c^	ND	8.1 ± 0.8^ab^	187 ± 17^a^
Hass Spain	62.3 ± 5.2^ab^	1.8 ± 0.2^a^	1.6 ± 0.05^a^	1.2 ± 0.09^ab^	ND	9.0 ± 1.0^ab^	76 ± 5^a^
Hass Perú	78.4 ± 9.8^b^	0.7 ± 0.4^a^	1.8 ± 0.08^a^	1.7 ± 0.6^abc^	ND	18.7 ± 0.6^c^	102 ± 10^a^
Hass Brazil	33.5 ± 8.4^a^	0.7 ± 0.3^a^	1.3 ± 0.2^a^	1.8 ± 0.4^abc^	ND	9.4 ± 0.5^b^	47 ± 8^a^

(**A**) Per 100 g fresh weight and (**B**) per g dry weight (DW). Data, which were obtained using the mesocarp of fruits in their optimum stage of ripening, show the mean of *n* = 4 fruits. Lower case letters (a–d) indicate differences between avocado varieties when *p* < 0.05. Trace amounts of δ-tocopherol and α-tocotrienol could not be properly quantified and are not shown here. T, tocopherol; TT, tocotrienol; PC-8, plastochromanol-8; ND, not detected.

## Supplementary Materials

The following are available online at https://www.mdpi.com/2076-3921/9/5/403/s1, Figure S1: Variations in the contents of tocochromanols during long-term (10 d) storage of “Bacon” avocados at cold temperatures (4 °C), Figure S2: Variations in the contents of chlorophylls, chlorophyll a/b ratio and the extent of lípid peroxidation (estimated as the contents of lípid hydroperoxides and malondialdehyde, as indicators of primary and secondary lípid peroxidation, respectively) during long-term (10 d) storage of “Bacon” avocados at cold temperatures (4 °C).
