# Supplementary material for: Identification of a New Variety of Avocados (Persea americana Mill. CV. Bacon) with High Vitamin E and Impact of Cold Storage on Tocochromanols Composition
Source: Antioxidants (Basel). 2020 May 9;9(5):403. doi: 10.3390/antiox9050403 (PMC7278777; doi:10.3390/antiox9050403)

SUPPLEMENTAL INFORMATION

Figure S1. Variations in the contents of tocochromanols during long-term (10d) storage of "Bacon" avocados at cold temperatures (4ºC). Data represents the mean ± SE of n=18 fruits. Lower case letters indicate differences between times when $P < 0.05$.

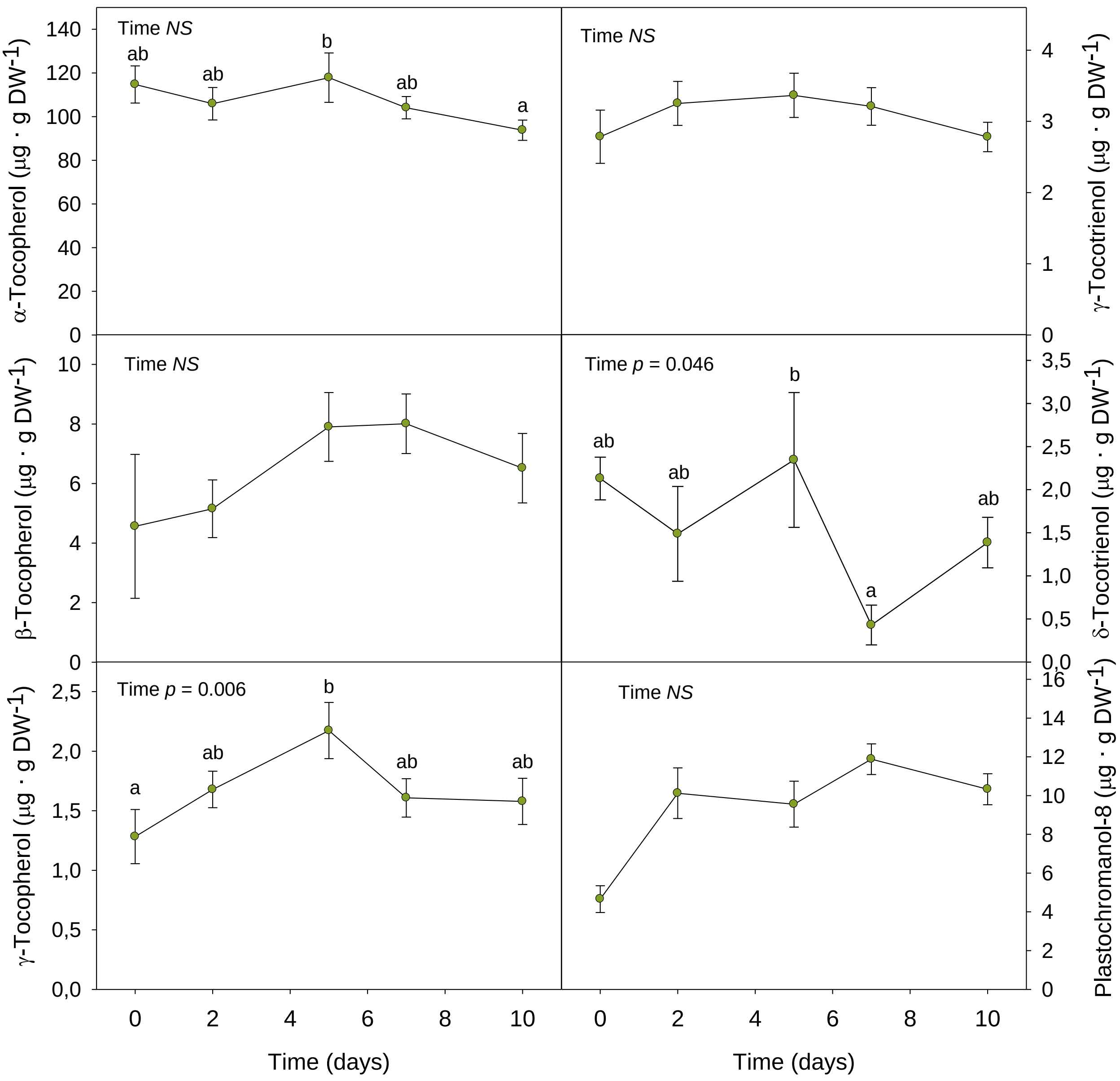

Figure S2. Variations in the contents of chlorophylls, chlorophyll a/b ratio and the extent of lípid peroxidation (estimated as the contents of lípid hydroperoxides and malondialdehyde, as indicators of primary and secondary lípid peroxidation, respectively) during long-term (10d) storage of “Bacon” avocados at cold temperatures (4ºC). Data respresent the mean ± SE of n=18 fruits. Lower cas letters indicate differences between times when $P < 0.05$.

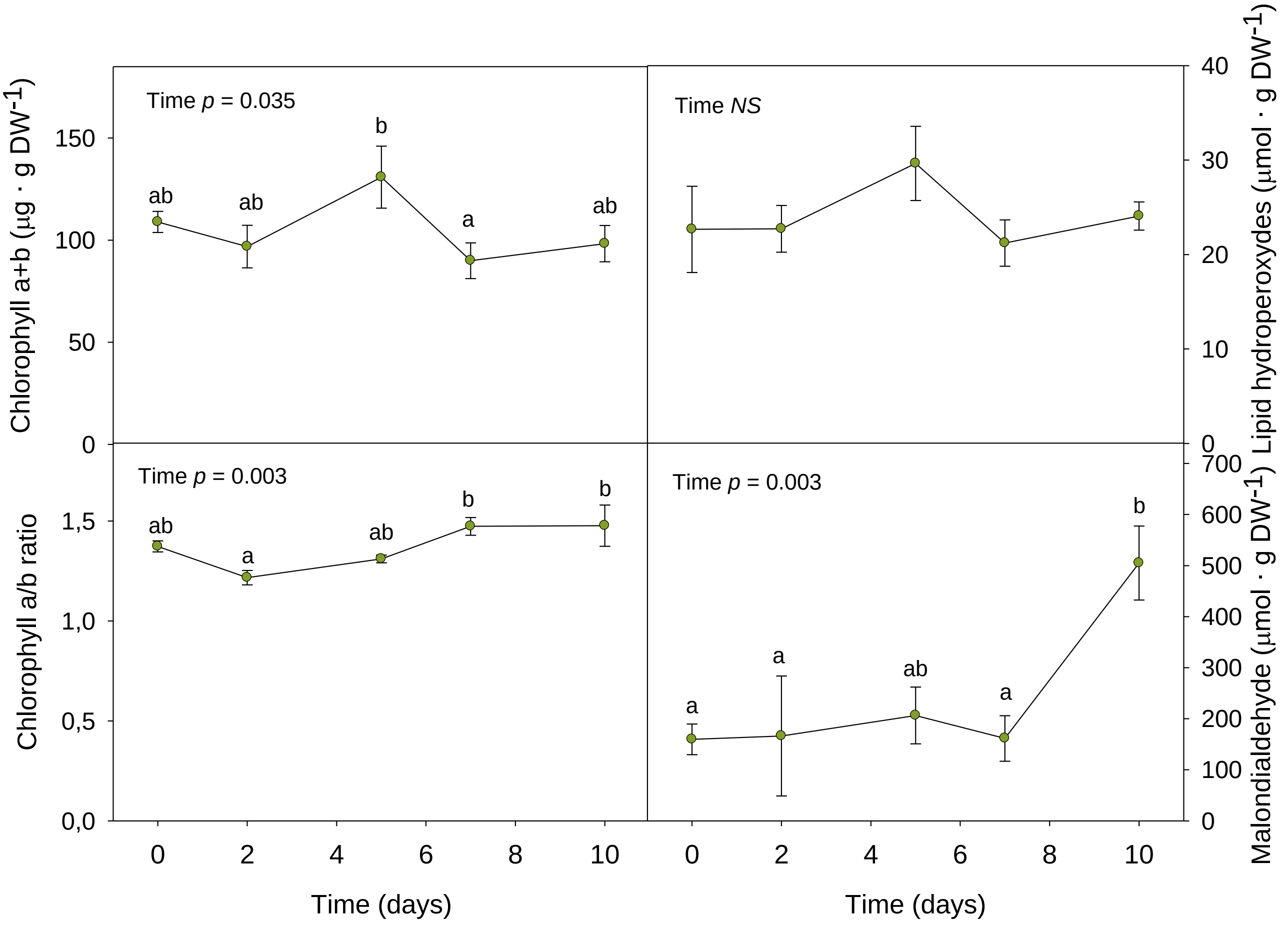

Supplement: Supplementary file 1 [file antioxidants-09-00403-s001.pdf]
